# Importance of customized (task oriented) software tools for biomedical applications

**DOI:** 10.6026/97320630016030

**Published:** 2020-01-15

**Authors:** Haseeb A Khan

**Affiliations:** 1 Department of Biochemistry, College of Science, King Saud University, Riyadh 11451, Saudi Arabia

## Abstract

Biomedical scientists and clinicians often require customized and user-friendly software tools due to the unavailability of specific software, complexity of existing software or high cost of commercial software. This editorial provides a short update on recently published task-oriented biomedical software tools. The novelty and applications of some customized software tools including CALCFISHER, CALCNTCP, CALCDOSE, and SCEW are also summarized in this editorial.

## Background:

Since the inception of computer technology, its applications flourished in multiples areas including biomedical sciences. Although the hardware (physical machinery) of computers are more or less similar, the variety of computer applications in different areas such as biology, medicine, engineering, aviation, weather forecast, etc. are governed by specific software (programming language) tools. The impact of computer application on biomedical sciences has been so enormous that it resulted in the emergence of a new field namely, bioinformatics. Even the scope of bioinformatics has now extended form gene sequence analysis and molecular docking to more diverse applications such as drug safety, survival analysis, metabolomics, imaging, gene expression, gene editing, etc. The term ‘big data’ is a consequence of the omics technologies and its analysis by machine learning has changed the climate of thought in biomedical sciences, shifting the demography of expertise and culminating in a new role of ‘data scientist’ [1]. In post-genomic era, digital health is yet another important emerging field based on utilizing the essences of internet, mobile apps and computer programming. Digital health is mainly the convergence of digital technologies with healthcare and society for enhancing the efficiency of healthcare delivery and making medicine more personalized and precise [2].

The advent of user-friendly programming languages and open source platforms has revolutionized the development of simple and efficient customized software tools for solving specific biomedical problems [3]. Souza et al. [4] have developed and validated the INVESALIUS NAVIGATOR as the first free, open-source software for image-guided transcranial magnetic stimulation (TMS) navigation, compatible with multiple tracking devices. INVESALIUS NAVIGATOR might contribute to improving spatial accuracy and the reliability of techniques for brain interventions by means of an intuitive graphical interface. To fulfill the need of a suitable tool that will measure data completeness of patient records, a data completeness Analysis Package (DCAP) has been developed with the major components including concept mapping, comma separated values (CSV) parsing, and statistical analysis [5]. DCAP examines patient records and generates statistics that can be used to determine the completeness of individual patient data as well as the general thoroughness of record keeping in a medical database. de Man and Limbago [6] presented an easy-to-use Sequence Search Tool for Antimicrobial Resistance [SSTAR]with a graphical userinterface that enables the identification of known antimicrobial resistance (AR) genes from whole genome sequences (WGS) and has theunique capacity to easily detect new variants of known AR genes, including truncated protein variants. GENE ANALYTICS is a comprehensive and easy-to-apply gene setanalysis tool for rapid contextualization of expression patterns and functional signatures embedded in the post-genomics big data domains, such as next generation sequencing (NGS), RNAseq, and microarray experiments. GENE ANALYTICS' differentiating features include in-depth evidence-based scoring algorithms, an intuitive user interface and proprietary unified data [7]. Khan [8-10] has developed Microsoft Excel Add-ins for color-coded visualization, filtering and comparison of microarray gene expression data and prediction of gene signatures by filtering the over- and under-expressed genes.

Sun et al. [11] have reported doctor-patient communication software that may help interns to improve their communication skills. CRCODER is an interactive web application to support clinical decision making based on electronic health data using joint modelling to generate an individual's probability of concurrent risk [12]. Recently, Stecher et al. [13] have published an updated version of the popular MEGA (Molecular Evolutionary Genetics Analysis) software. This new version eliminates the need for virtualization and emulation programs previously required to use mega on Apple computers. MEGA for MACOS utilizes memory and computing resources efficiently for conducting evolutionary analyses on Apple computers [13]. Falzon et al. [14] developed a software tool, classify me, for the automated identification of animal species from camera trap images. Classify me identifies animals and other objects in images, provide a report file with the most likely species detection, and automatically sort the images into folders corresponding to species categories.

The software CALCFISHER is a convenient tool for comparing the significance level between the two groups using Fisher's exact test (FET) [15]. The computations involved in FET are extremely tedious and time consuming due to multi-step factorial calculations after the construction of numerous 2x2 tables depending on the smallest cell value. The standard FET formula looks simple and straightforward and can be used for very small number of frequencies. However, if the sample size is larger, the hand-held scientific calculator fails to calculate the P value. Even with the help of a computer, the regular formula can only be used for up to a total of 113 subjects, beyond that the output of factorial computations exceeds the range of a computer (1.79E308; in simple language 306 zeros after 179). To resolve this issue, Khan [15] invented a log-based formula and used it for developing the software CALCFISHER, for computing the Fisher’s exact probability. The CALCFISHER software is extremely useful in medical research for statistical comparison of two groups. This is the only software currently available that can compute Fisher's exact P values for >10,000 samples [15] and has been utilized in several studies [16-22].

The software is a simple, user-friendly and time-saving tool for quick and accurate computation of normal tissue complication probability (NTCP), which is a useful parameter to determine the possibility for normal tissue to be adversely affected by radiation therapy [23]. This software is potentially useful in helping the clinicians for quick evaluation or optimization of the radio therapy treatment plans. Although the methodology of NTCP calculation is straightforward, the main difficulty arises while computing the normal distribution error function for cumulative probability. Hence, the computation of NTCP tends to be very complex or practically impossible without the aid of a suitable computer program or statistical tables. CALCNTCP software is extremely useful for multiple computations in a short time. Several investigators have reported the use of this software [24,25].

The software CALCDOSE was developed using Visual Basic language and aimed for drug dosage conversions using metabolically active mass (MAM) of animals [26]. In fact, small animals require large dosages of a drug (per kilogram body weight) as compared to big animals or humans for similar pharmacological effects. These variations in dose requirement are related to varying metabolic activities of animals with large differences in their body weights. However, there is a remarkable consistency of the daily expenditure of energy expressed permetabolically active mass (MAM), irrespective of the body weight of animals. The lean body mass, which accounts for about 75% of the body weight, contains all the body protein and that mass is considered as metabolically active. Mathematically, MAM is equal to body weight raised to 0.75 power, and holds true for freely feeding animals of different sizes and weights. Pharmacologists often face the problem of finding an optimum reference dosage for their initial pilot studies whereas the CALCDOSE software can be effectively used to calculate human equivalent dose of a drug that has been pre-tested in experimental animals [26].

Khan [27] has reported a simple and novel method for visualization of experimental gastric lesions by direct scanning of stomach samples and their quantification by computer-assisted image analysis. The manual assessment and quantitation of experimentally induced gastric lesions is a problematic and error-prone task due to their predominantly multiple and irregularly shaped occurrence. Direct scanning of stomach samples, sandwiched in a transparent plastic folder, was demonstrated to be a simple, cost-effective, rapid, and efficient protocol for morphologic evaluation of experimental gastric lesions. The output images of scanned stomach samples appeared to be superior to scanned photographs, and ready to be used for quantitative assessment of mucosal injury using the image analysis software. This protocol has been successfully applied by other investigators for quantification of gastric lesions [28] as well as experimental colitis [29].

Considering the familiarity of clinicians and biomedical scientists with Excel, an add-in program, survival curves in Excel work sheet (SCEW),was developed for easy creation of survival curves directly in Excel worksheets [30].Although Excel offers a wide range of graphical applications including line graph, scatter graph, bar graph and pie chart it lacks the facility of survival curves. However, with the help of SCEW, the step-wise survival curves can directly be created in an Excel worksheet. SCEW utilizes Excel’s worksheet formula function for instant data configuration and the modified data are used to construct survival curve with the help of a macro subroutine. This automation renders the entire methodology more convenient, faster and error-free as compared to manual procedure. The use of SCEW has been reported by other investigators in their studies [31,32].

## Conclusion:

Customized software tools are often developed by scientists and academicians for utilization by the global scientific community. These software programs efficiently fulfill the specific requirements and perform the task efficiently. Moreover, these tools tend to be user-friendly and often available free of cost. The development and validation of specific software tools do not require expensive equipment or laboratory space but time, mental power and dedication. Unfortunately, there is a dearth of financial support in this sector and therefore the institutions and government must encourage and financially support the projects related to the development of customized software tools for biomedical applications.
